# Seasonal variation in trans-oceanic flight and island use in a migratory falcon

**DOI:** 10.1093/beheco/arag094

**Published:** 2026-08-03

**Authors:** Meixu Chen, Duarte S Viana, Jordi Figuerola, Laura Gangoso, Wouter M G Vansteelant

**Affiliations:** BirdEyes Center for Global Ecological Change at the Faculties of Science and Engineering and Campus Fryslan, University of Groningen, Wirdumerdijk 34, 8911 CE Leeuwarden, the Netherlands; GELIFES, Faculty of Science and Engineering, University of Groningen, Linnaeusborg (5th floor), Nijenborgh 7, 9747 AG Groningen, the Netherlands; Department of Conservation Biology and Global Change, Estación Biológica de Doñana (EBD-CSIC), Calle Américo Vespucio, 26, 41092 Seville, Spain; Department of Conservation Biology and Global Change, Estación Biológica de Doñana (EBD-CSIC), Calle Américo Vespucio, 26, 41092 Seville, Spain; Centro de Investigación Biomédica en Red de Epidemiología y Salud Pública (CIBERESP), Avenida de Monforte de Lemos 3-5, Pabellón 11, Planta 0, 28029 Madrid, Spain; Department of Conservation Biology and Global Change, Estación Biológica de Doñana (EBD-CSIC), Calle Américo Vespucio, 26, 41092 Seville, Spain; BirdEyes Center for Global Ecological Change at the Faculties of Science and Engineering and Campus Fryslan, University of Groningen, Wirdumerdijk 34, 8911 CE Leeuwarden, the Netherlands; GELIFES, Faculty of Science and Engineering, University of Groningen, Linnaeusborg (5th floor), Nijenborgh 7, 9747 AG Groningen, the Netherlands; IBED, University of Amsterdam, Sciencepark 904, 1098 XH Amsterdam, the Netherlands

**Keywords:** barrier crossing, bird migration, stepping stones, stop-over behavior, weather

## Abstract

Wind conditions play a major role in determining how birds negotiate ecological barriers during long-distance migration. Migrants typically minimize flights over barriers in opposing winds, while crossing the same barriers directly in supportive winds. Nevertheless, even in supportive conditions, direct barrier-crossings may be associated with an elevated risk of fatigue. At the same time, barrier-crossings may be facilitated by the availability of steppingstones. We aimed to understand how seasonal ocean-crossings of Eleonora's falcons (*Falco eleonorae*) compare in terms of wind support, flight effort, and the use of remote islands. To do this we combined over a decade of GPS-tracking data from 19 individuals (2012 to 2022) with wind data from an atmospheric reanalysis model. Despite far greater wind support in spring, falcons achieved similar ground speeds in both seasons, because they flew at much lower airspeeds in spring (8.8 ± 1.7 m/s) than in autumn (13.2 ± 2.6 m/s). Overall, it took falcons twice as many flight hours to complete a 40% greater air distance in spring compared with autumn. More islands are available along the spring route, and 57.9% of falcons used an island during at least 1 spring crossing, compared with 21.1% in autumn. In spring, island use increased with weaker wind support, with falcons spending the night on islands, and apparently maximizing daytime flight by initiating ocean-crossings at dawn. Overall, wind conditions only partially offset the seasonal difference in falcons' crossing distance and effort. Isolated islands may be an overlooked factor explaining the configuration of falcons' ocean-crossing corridors.

## Teaser

How do falcons cope with long ocean crossings? Using a decade of GPS tracking, we analyse how Eleonora’s falcons use seasonal winds and remote islands to cross the Indian Ocean. Wind only partly offsets seasonal differences in crossing distance and effort. Falcons adjust flight speed and island use in relation to wind conditions, shaping how they complete ocean crossings.

## Introduction

Long-distance migration often requires individuals to overcome ecological barriers; ie landscapes far outside a species' habitual niche that offer limited opportunities for resting and refueling, or characterized by extreme environmental conditions (Alerstam 1993; Lathouwers et al. 2022; Newton 2023). For migrant landbirds, the riskiest barriers are large water bodies where they cannot land and thus face a risk of drowning in case of inclement weather or fatigue. As such, birds often circumvent large water bodies entirely, or minimize the time and distance flown over water by crossing water bodies at their narrowest points (eg sea-straits). The aversion for crossing water bodies is especially pronounced in obligate-soaring migrants that incur a disproportionate increase in flight costs when atmospheric conditions force them to resort to active, flapping flight—which is often the case over open water (Duriez et al. 2018; Blas et al. 2020; Mellone 2020; Nourani et al. 2020, 2021; Pekarsky et al. 2024). More broadly speaking, the extent to which landbirds avoid water bodies depends in large part on prevailing winds: birds are more likely to cross directly in supportive than adverse winds (Gill et al. 2014; Vansteelant et al. 2017a; Becciu et al. 2020; Norevik et al. 2020; Literák et al. 2021; Bathrick et al. 2024).

Wind support has such a strong effect on flight costs (Liechti 2006) that prevailing winds explain the seasonal configuration of global aerial flyways in general (Kranstauber et al. 2015; Vansteelant et al. 2017a), and especially in relation to ecological barriers (Mellone et al. 2016; Shamoun-Baranes et al. 2017; Newton 2023). Indeed, numerous migrants exhibit seasonal loop migrations because prevailing winds (or other weather factors) permit them to cross a barrier directly in one but not the other season (Becciu et al. 2020; Nourani et al. 2016, 2021; Vansteelant et al. 2017c, 2021). Nevertheless, crossing a large expanse of open water in supportive winds may still involve a greater sustained effort, and a relatively greater risk of fatigue, than crossing a shorter distance against the wind. To compare seasonal barrier-crossings in terms of flight effort, it is important to account for wind effects and consider the birds' instantaneous effort in terms of airspeed (Liechti 2006), and cumulative effort over the full duration of the flight in terms of air distance (ie the distance achieved by the bird relative to the air).

Another factor that can facilitate barrier-crossings is the availability of isolated patches of more benign habitat. For example, recent tracking studies have shown that some Afro-Palearctic migrants make multiday stop-overs while crossing the “sea of sand” of the Sahara Desert, suggesting that (in some years) certain parts of the desert actually do offer rest and foraging opportunities (Strandberg et al. 2010; Monti et al. 2024). Shorebirds crossing the “sea of green” of the Amazon Basin were found to use riverbanks and lakes in unexpectedly high numbers (Linscott et al. 2024), while forest patches serve as stepping stones for landbirds traversing the Corn Belt in the United States (Guo et al. 2025). Analogously, it seems only logical that ocean-crossing landbirds may use islands, as they appear to do in the Central Mediterranean and along island chains in eastern Asia (Agostini et al. 2004; Bildstein 2006; Nourani et al. 2018). Yet, it is unclear if and how remote oceanic islands are used by ocean-crossing landbirds more generally, and if so, whether they are used on a regular basis as stepping stones, or more opportunistically as “emergency stop-overs” (Shamoun-Baranes et al. 2010). Inclement weather during sea-crossings can result in mass mortality events (Newton 2025) and so-called fall-outs whereby huge numbers of birds land on islands, offshore structures and ships to sit through adverse weather (Ronconi et al. 2015; Shamoun-Baranes et al. 2017; Sarà et al. 2023). It is conceivable that (remote) islands play an underappreciated role as stepping-stones or stop-overs under adverse conditions in many trans-oceanic migrations.

**Figure 1:**
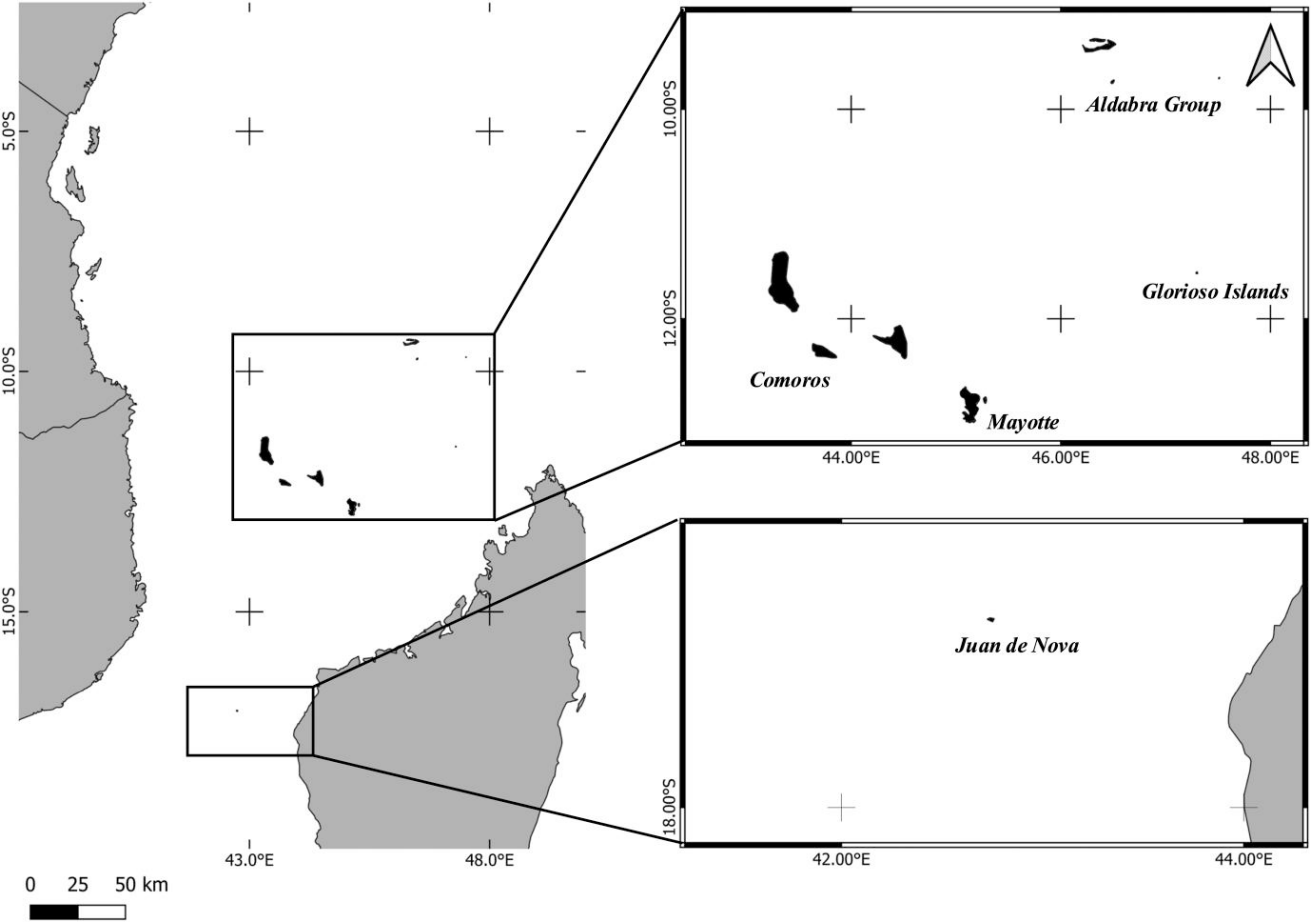
Study area in the Western Indian Ocean. Geographic extent of the study area includes the Aldabra Group, Glorioso Islands, Mayotte, the Comoros, and Juan de Nova. Insets show magnified views of the northern and southern island clusters. Shaded regions indicate surrounding landmasses, including north Madagascar and the eastern African coast. Crosses indicate graticule intersections (latitude/longitude reference points). Country boundaries were obtained from Natural Earth (public domain).

In this study, we aimed to better understand how long-distance migrant Eleonora's falcons *Falco eleonorae* cope with large seasonal differences in wind support and island availability when crossing the Indian Ocean on the way to and from their Malagasy nonbreeding grounds. It has been well established that falcons from throughout the species' breeding range spend the nonbreeding season in northern Madagascar (Kassara et al. 2017), and they cross the ocean along distinct corridors in autumn and spring. More specifically, they cross the Indian ocean at its narrowest point near the Mozambique Channel (∼420 km) in opposing autumn winds (López-López et al. 2009, 2010). By contrast, they cover at least twice that distance (and often much more) by crossing directly from northern Madagascar to mainland East Africa in supportive spring winds (Gschweng et al. 2008; López-López et al. 2010; Mellone et al. 2013; Hadjikyriakou et al. 2020; Vansteelant et al. 2021). We also know that individual falcons exhibit highly flexible spring routes over the Indian Ocean depending on annual variations in wind conditions (Mellone et al. 2011; Vansteelant et al. 2023). However, it remains unclear to what extent the supportive winds in spring compensate for the additional distance flown over the ocean in spring compared with autumn, and how falcons adjust their flight times and speeds (ie airspeeds) to prevailing winds. Moreover, there are a number of small islands, archipelagos, and atolls along the way that may serve as stepping stones (Nourani et al. 2018) or emergency stop-over sites (including Comoros, Glorioso Islands and Aldabra group, Fig. 1). There is anecdotal evidence that falcons sometimes stop-over (tracking) or perish on these islands (ring recoveries) (Gschweng et al. 2008). But it is unknown how regularly Eleonora's falcons use islands, and if so, under what environmental conditions, for how long, and what is the function of island visits (ie resting, foraging or both).

Our study leverages high-resolution GPS-tracking data from Eleonora's falcons studied at the species' westernmost population on the Canary Islands. Like conspecifics from other parts of the breeding range, falcons from our study population use distinct seasonal corridors over the Indian Ocean: they minimize ocean-crossing distance and reduce headwind resistance by crossing the Mozambique Channel near its narrowest point in autumn, and they fly almost perfectly downwind during a protracted ocean-crossing directly from Madagascar in spring (Vansteelant et al. 2021). We (i) quantify seasonal differences in ocean-crossing distance and duration, and the use of islands, (ii) quantify the diel timing of trans-oceanic flights (incl. timing of take-off and landings from/on the mainland and islands), and (iii) assess diel activity patterns of falcons on islands. Next, we (iv) test for seasonal differences in instantaneous and cumulative wind support (ie average tailwind speed and overall wind displacement), and falcons' instantaneous and cumulative flight effort over the ocean (ie average airspeeds and air distances), and finally, (v) test whether wind conditions experienced by falcons over the ocean affect the likelihood of them using islands in either season.

We know from previous research that falcons are more likely to encounter islands along their spring ocean-crossing corridor than over the Mozambique channel in autumn (Vansteelant et al. 2021, Fig. 1), and we therefore expect that a greater proportion of individuals used islands during spring than autumn crossings. We also know that falcons receive greater wind support during spring ocean-crossings, and here we expect that this allows falcons to cross at lower airspeeds, ie to reduce instantaneous flight cost compared with autumn. At the same time, while wind support should compensate for much of the additional ocean-crossing distance in spring, we expect falcons still make a greater cumulative flight effort (ie air distance) over the ocean in spring, and that they are more likely to use islands as stop-overs when encountering relatively weak wind support.

## Materials and methods

### Study area

This study investigates the migratory patterns of Eleonora's falcons across the Indian Ocean and Mozambique Channel, including the potential role of islands located 100 to 400+ km offshore from East Africa or Madagascar. Along these routes, several island groups—including the Seychelles (particularly the Aldabra Group), the Glorioso Islands, Mayotte, the Comoros (Grande Comore, Mohéli, and Anjouan), and Juan de Nova can be reached and, in theory, could function as stepping stones or emergency stop-over sites for falcons while they are far offshore (Fig. 1).

### Falcon tracking

We tracked Eleonora's falcons from the species' westernmost breeding population on the Canary Islands (Gangoso and Figuerola 2019). Adult falcons were caught at the nest or at a drinking pool on the islet of Alegranza, which holds about 127 breeding pairs representing just under half of the Canarian population (Gangoso et al. 2020, 2015). UvA-BiTS GPS loggers, weighing 7.5 g, were attached to falcons using a Teflon body harness (Gangoso et al. 2020). Data was downloaded through a locally constructed network of antennas, and we obtained migration data for 19 adult individuals that returned to Alegranza after completing their migration cycle to and from Madagascar. More details on catching and tagging procedures, return and data retrieval rates are provided by Vansteelant et al. (2021). The devices recorded positions at varying intervals depending on battery levels, and for the ocean-crossing flights the temporal resolution of the data varied from 0.1 to 10 min intervals for 99% of all data points.

### Identifying ocean-crossings and island visits

We identified the start and end points of crossings based on their geographic location. More specifically, we selected the last/first location on Tanzania, Kenya or Mozambique as the start of autumn and the end of spring crossings, respectively. Analogously, we selected the first/last point on Madagascar as the end of autumn and the start of spring crossings, respectively. In total, our dataset comprised 83 sea-crossing events by 19 individuals across 10 annual cycles between 2012 and 2022, including 42 autumn and 41 spring sea-crossing events.

Islands near (ie < 50 km from) the East African and Malagasy shores were considered as part of the mainland or Malagasy landmass for the purpose of delimiting the start and end of sea-crossing segments. In other words: in our analyses of how falcons use islands as possible stepping-stones or stop-overs, we focus exclusively on those islands and atolls that are situated at least 100 km (Juan de Nova) and in most cases >250 km from the nearest major landmass (see “Study area”). To ensure even the smallest islands were included, we created a custom shapefile by tracing island/atoll contours in Google Earth v7.3.6.1020 (imagery 12 March 2024).

### Estimating wind support

All GPS positions were annotated with wind data from the NOAA-NCEP Reanalysis II model (resolution 6 h and 2.5°× 2.5°; [Kalnay et al. 1996; Kemp et al. 2010]). Based on the falcons recorded flight altitudes, we decided to use the u- and v-wind components at the 925 mb pressure level, corresponding to approximately 750 meters above sea level (Figure S1). Using basic trigonometry we then converted the u- and v-wind components to wind speed and direction, calculated the speed of tail-/headwind and sidewinds relative to the birds' travel directions (Vansteelant et al. 2021), and finally the birds' airspeed and air distance (as the product of time and airspeed) from each GPS-position to the next. In addition, to visualize seasonal wind fields over the Indian Ocean, we extracted u- and v-wind estimates for each model run during the autumn and spring ocean-crossing periods, and calculated the average wind strength and speed for every node in the model grid. The reanalysis models have a much coarser temporal resolution than the tracking data, and are known to be less accurate in parts of the global south (Kalnay et al. 1996). However, the models can be expected to capture true spatial and temporal patterns in wind strength and direction between and within seasons, and have been used in numerous other studies of migratory flight behavior (Shamoun-Baranes et al. 2017), including previous work on the species studied here (Vansteelant et al. 2021).

### Quantifying seasonal ocean-crossing metrics

After extracting ocean-crossings and annotating them with island and wind data, we calculated several behavioral and wind support metrics. For each crossing, we calculated (i) total crossing distance, (ii) total crossing duration, including time spent on islands; (iii) time spent on islands as the sum of time intervals across all positions classified on islands; (iv) the number of distinct islands visited during the crossing. Next, for each crossing we averaged the (v) tailwind speed, (vi) ground speed, (vii) airspeed across all flight locations, and calculated the (viii) cumulative wind displacement (ie the distance over which a falcon was pushed forward or backward along its travel direction with respect to the ground), and the (ix) cumulative air distance (ie the distance achieved by a falcon with respect to the air, which is greater than ground distance when confronting headwinds, and lower than ground distance when flying with strong tailwinds). Airspeed and air distance serve as proxies for instantaneous and cumulative energy expenditure during active flapping flight over the ocean (Liechti 2006; Pennycuick 2008). Given the consistently high resolution of our data, we expect that the accuracy of our estimates for airspeeds and air distances mainly depends on the accuracy of wind estimates in the reanalysis model.

### Diel timing of ocean-crossings and activity patterns on islands

We used the suntools package (Bivand and Luque 2024) to determine the timing of sunrise, sunset and solar noon on each location and day. All positions before civil sunrise and after civil sunset were classified as night locations. We then assessed diel timing of ocean-crossings by calculating at what “solar time” falcons departed or completed the ocean-crossing, and during island visits. We quantified peaks in departure and arrival frequencies relative to (civil) sunrise and sunset, and assessed diel activity during island visits using ground speeds to explore whether falcons were active or resting on islands during night and day. These activity measures can be understood as a proxy for the function of island visits, ie, whether they are used primarily for resting, foraging, or both.

### Statistical analyses

All statistical analyses and data visualization were conducted in R version 4.3.1 (R Core Team 2023), except for Fig. 1 which was made in QGIS (v.3.30.2; QGIS Development Team 2023). To evaluate the statistical significance of seasonal differences in aforementioned flight, island use, and wind support metrics we utilized a Generalized Linear Mixed Modelling (GLMM) approach. We included a fixed effect for season, to estimate the average difference between autumn and spring estimates, and random intercepts for individual birds to account for repeated measurements of the same individuals. Most of the variables satisfied the assumption of normally distributed residuals. For the variable “Island Count”, an integer variable, residuals best fitted a Poisson distribution. In cases where the estimation of random individual intercepts in mixed models resulted in singular fits we instead adopted generalized linear models (GLM).

Next, we aimed to analyze how departure location (specifically latitude) and wind conditions experienced over the ocean affected the probability of a falcon using an island. We quantified wind support during the “first half” of the ocean-crossing because falcons typically encounter islands around the mid-way point of seasonal ocean-crossings. We defined the “first half” as the moment of initial take-off until (a) the first fix recorded over an island or, (b) in cases where falcons did not pass over an island, a hand-drawn boundary representing the approximate half-way point along the autumn and spring ocean-crossing corridors, respectively. Following this segmentation we calculate the mean head-/tailwind that falcons experienced during the first half of each sea-crossing. We again used GLMMs to implement a logistic regression for each season separately, with the probability of using an island (0, 1) as response variable, the average wind support during the first half of the crossing and departure latitude as fixed effects, and allowing for randomly varying intercepts between individuals to account for nonindependence among repeated measurements of the same individuals. Finally, to help assess the way in which falcons use islands, we used GLMMs with a beta residual distribution to test whether the proportion of time spent resting was correlated with the time spent on the island at night or by day and the total duration of island visits. In all cases where the inclusion of random individual intercepts in GLMMs resulted in singular fits, we corroborated effects using GLMs instead.

## Results

### General description of seasonal ocean-crossings

**Figure 2:**
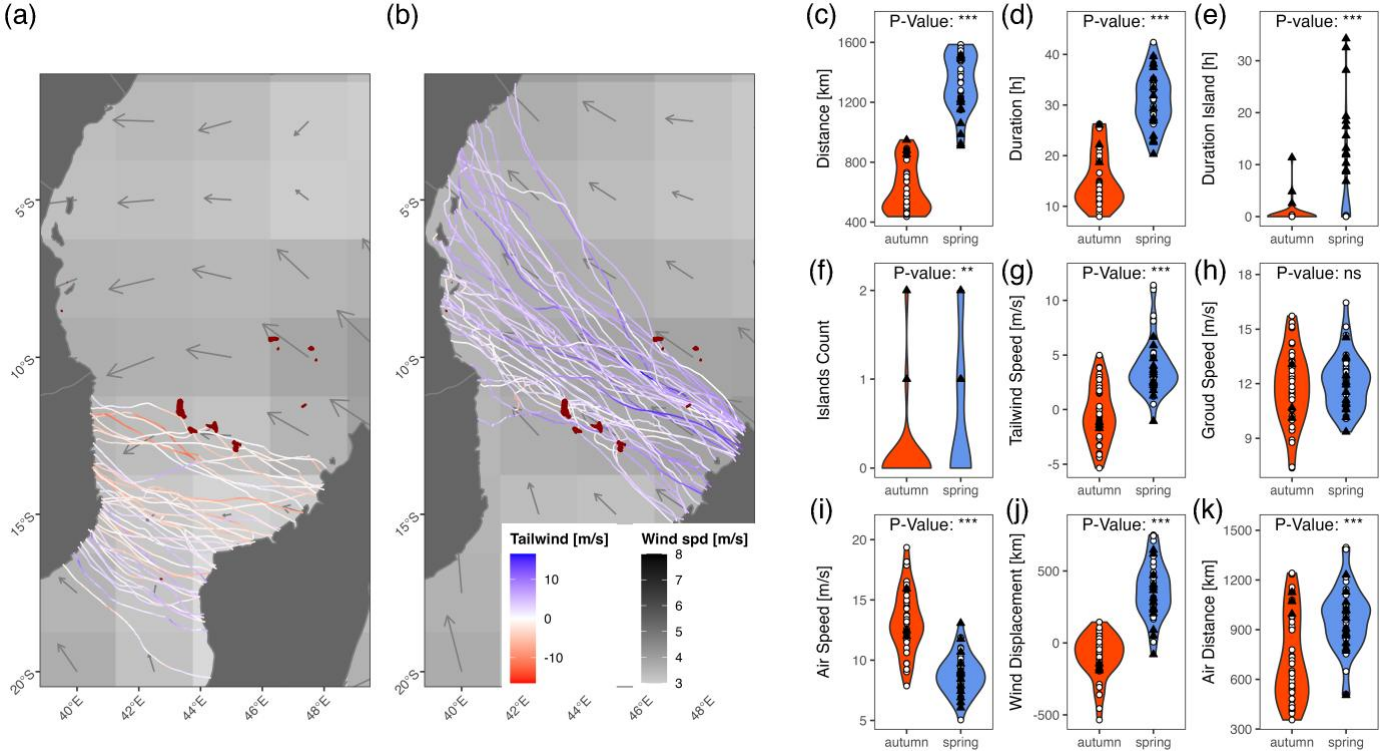
Seasonal comparison of eleonora's falcons' ocean-crossings between east Africa and Madagascar. (a, b) Ocean-crossing trajectories are shaded according to tailwind support along the falcons' realized travel direction, as indicated by the tailwind scale. Background shading and arrows indicate average wind conditions over the ocean in 2.5°×2.5 °cells, with darker shading and longer arrows indicating stronger winds. Remote islands situated within the falcons' seasonal crossing corridors are highlighted. shown in dark red. (c–k) Violin plots showing the seasonal distribution of ocean-crossing parameters, based on the first recorded crossing of each individual: (c) total distance flown over the ocean, (d) total crossing duration, (e) time spent on islands, number of islands used, (g) average tail-/headwind strength along the falcons' realized tracks, (h) average ground speeds, (i) average airspeeds (j) cumulative wind displacement, and (k) total air distance flown over the ocean. Points indicate values for individual crossings, with circles indicating crossings on which falcons did not use any island, and triangles indicating crossings on which falcons used at least one island. Labels indicate significance of seasonal differences according to pairwise t-tests (ns: not significant, *: 0.05 < *P* < 0.01; **: *P* < 0.01; ***: *P* < 0.001).

Falcons started their autumn ocean-crossings on 14th November ± 5.3 days, starting from the coast of Mozambique or Tanzania to cross the Mozambique Channel near its narrowest point (Fig. 2a). The ocean-crossing corridor was bordered by the islands of Mayotte and the Comoros to the north. Falcons typically experienced headwinds during autumn crossings, though some birds that crossed south of the narrowest point of the Channel experienced weak-moderate tailwinds (Fig. 2a, note north-south gradient in track colors). By crossing south of Mayotte and the Comoros, the falcons effectively avoided an area with much stronger headwinds north of the islands (Fig. 2a: note longer wind arrows and darker background shading).

Falcons started their spring ocean-crossings on 10th April ± 7.5 days, departing from northern Madagascar in a northwestern direction to mainland East Africa (Fig. 2b). The transoceanic spring corridor was bordered by the islands of Mayotte and the Comoros to the south, and the Seychelles' Aldabra group to the north (Fig. 2b). In general, falcons enjoyed moderate-strong tailwinds (Fig. 2b, note strong dominance of blue colored tracks). The spring ocean-crossing corridor largely overlapped with the area where prevailing (south-) easterlies were strongest (Fig. 2b, note background shading is darkest where falcons cross in spring).

### Seasonal ocean-crossing distance, duration, and island use

Autumn ocean-crossings spanned a distance of 400 to 1,000 km (Fig. 2c, Table S1), which falcons typically completed in less than a day (15.3 ± 5.4 h, Fig. 2d, Table S1). Spring crossings were significantly longer at 800 to 1,600 km (Fig. 2c, Table S1), and typically took twice as long—usually more than a day (31.0 ± 5.2 h, Fig. 2d, Table S1) to complete. The difference in crossing duration was associated with a greater amount of time spent on greater number of islands in spring (Fig. 2e–f, Table S1). This was due to a greater proportion of individuals using islands in spring (Table 1) as well as island visits taking longer in spring (Tables 1 and 2). Island use was not the norm in either season, as >60% of the ocean-crossings were completed in nonstop flight. However, 68% of the falcons did use an island in at least one of the years they were tracked, and the frequency of island use was much higher in spring (57% of falcons) compared with autumn (15% of falcons, Table 1). The islands used by most individuals were Comore Grande (26% of falcons) and Moheli (21% of falcons) in the Comoros, and the island of Mayotte (21% of falcons) (Table 2).

**Table 1:** Summary statistics including seasonal sample sizes, ocean-crossing distance and duration, number of islands used, and time spent on islands, corresponding to Fig. 2c-f.

Falcon iD	Season	Number of trips	No. of islands	Distance (km)		Duration (h)		Island duration(h)		Island count	
				Mean	SD	Mean	SD	Mean	SD	Mean	SD
**B1011**	**Autumn**	3	2	**869**.**6**	**160**.**8**	**21**.**7**	**1**.**5**	**0**.**8**	**1**.**5**	**0**.**7**	**1**.**2**
**B1011**	**Spring**	**2**	**2**	**1,233**.**3**	**48**.**5**	**46**.**0**	**24**.**2**	**17**.**1**	**24**.**2**	**1**.**0**	**1**.**4**
B1012	Autumn	3	0	729.9	75.6	25.7	0.0	0.0	0.0	0.0	0.0
**B1012**	**Spring**	**3**	**3**	**1,154**.**4**	**173**.**3**	**34**.**0**	**7**.**5**	**4**.**5**	**7**.**5**	**1**.**0**	**1**.**0**
B1013	Autumn	2	0	669.8	314.1	20.2	0.0	0.0	0.0	0.0	0.0
B1013	Spring	2	0	1,347.5	179.3	27.6	0.0	0.0	0.0	0.0	0.0
B1014	Autumn	4	0	490.2	57.9	11.6	0.0	0.0	0.0	0.0	0.0
B1014	Spring	4	0	1,519.2	82.5	33.4	0.0	0.0	0.0	0.0	0.0
B2048	Autumn	2	0	618.2	160.1	14.7	0.0	0.0	0.0	0.0	0.0
**B2048**	**Spring**	**2**	**1**	**1,445**.**9**	**103**.**5**	**41**.**1**	**12**.**3**	**8**.**7**	**12**.**3**	**0**.**5**	**0**.**7**
B2051	Autumn	2	0	667.1	285.2	15.8	0.0	0.0	0.0	0.0	0.0
**B2051**	**Spring**	**2**	**1**	**1,396**.**7**	**165**.**0**	**40**.**7**	**7**.**3**	**5**.**1**	**7**.**3**	**0**.**5**	**0**.**7**
B2337	Autumn	1	0	720.9	…	19.0	…	0.0	…	0.0	…
B2337	Spring	1	0	1,483.2	…	38.2	…	0.0	…	0.0	…
B2368	Autumn	2	0	599.3	99.5	13.4	0.0	0.0	0.0	0.0	0.0
**B2368**	**Spring**	**2**	**1**	**1,291**.**0**	**382**.**0**	**34**.**8**	**8**.**6**	**6**.**1**	**8**.**6**	**0**.**5**	**0**.**7**
B2378	Autumn	1	0	521.0	…	10.9	…	0.0	…	0.0	…
B2378	Spring	1	0	1,340.6	…	35.2	…	0.0	…	0.0	…
B2380	Autumn	1	0	582.8	…	14.4	…	0.0	…	0.0	…
B2380	Spring	1	0	1,335.8	…	28.0	…	0.0	…	0.0	…
B2391	Autumn	3	0	686.7	203.6	16.5	0.0	0.0	0.0	0.0	0.0
**B2391**	**Spring**	**3**	**5**	**1,160**.**7**	**196**.**0**	**55**.**9**	**7**.**2**	**26**.**4**	**7**.**2**	**1**.**7**	**0**.**6**
B2392	Autumn	3	0	660.2	172.6	13.9	0.0	0.0	0.0	0.0	0.0
**B2392**	**Spring**	**3**	**3**	**1,340**.**5**	**195**.**2**	**36**.**1**	**3**.**9**	**2**.**3**	**3**.**9**	**1**.**0**	**1**.**0**
B2394	Autumn	1	0	436.8	…	9.4	…	0.0	…	0.0	…
**B2394**	**Spring**	**1**	**2**	**1,516**.**8**	**…**	**46**.**4**	**…**	**8**.**9**	**…**	**2**.**0**	**…**
B2400	Autumn	4	0	479.0	29.2	10.5	0.0	0.0	0.0	0.0	0.0
**B2400**	**Spring**	**4**	**3**	**1,368**.**2**	**199**.**5**	**33**.**7**	**7**.**5**	**6**.**1**	**7**.**5**	**0**.**8**	**1**.**0**
**B2423**	**Autumn**	**3**	**2**	**611**.**2**	**263**.**7**	**17**.**5**	**6**.**5**	**3**.**8**	**6**.**5**	**0**.**7**	**1**.**2**
B2423	Spring	3	0	1,376.6	144.6	29.4	0.0	0.0	0.0	0.0	0.0
B2424	Autumn	2	0	540.6	38.9	10.6	0.0	0.0	0.0	0.0	0.0
**B2424**	**Spring**	**2**	**1**	**1,428**.**1**	**138**.**4**	**40**.**6**	**8**.**4**	**5**.**9**	**8**.**4**	**0**.**5**	**0**.**7**
**B2447**	**Autumn**	**1**	**1**	**858**.**4**	**…**	**27**.**0**	**…**	**4**.**8**	**…**	**1**.**0**	**…**
B2447	Spring	1	0	1,421.8	…	33.2	…	0.0	…	0.0	…
B2450	Autumn	1	0	507.6	…	13.3	…	0.0	…	0.0	…
B2450	Spring	1	0	1,149.1	…	26.3	…	0.0	…	0.0	…
**B2453**	**Autumn**	**3**	**1**	**662**.**9**	**174**.**6**	**14**.**7**	**0**.**2**	**0**.**1**	**0**.**2**	**0**.**3**	**0**.**6**
**B2453**	**Spring**	**3**	**1**	**1,320**.**5**	**194**.**4**	**34**.**6**	**11**.**1**	**6**.**4**	**11**.**1**	**0**.**3**	**0**.**6**

Bold rows indicate individuals that used at least one island in one of their recorded journeys.

**Table 2:** Island use by Eleonora’s Falcons during autumn and spring ocean-crossing.

Season	Island	Mean island stop-over duration (h)	No. of individuals	No. of trips
Autumn	Anjouan	0.11	3	3
Autumn	Mayotte	3.46	2	2
Autumn	Mohéli	10.38	1	1
Spring	Aldabra	10.27	3	3
Spring	Anjouan	18.07	1	1
Spring	Assomption	0.03	1	1
Spring	Grande Comore	11.23	5	6
Spring	Grande Glorieuse	8.54	2	2
Spring	Mayotte	11.37	4	6
Spring	Mohéli	8.62	4	4

### Diel timing of ocean-crossings and activity on islands

**Figure 3:**
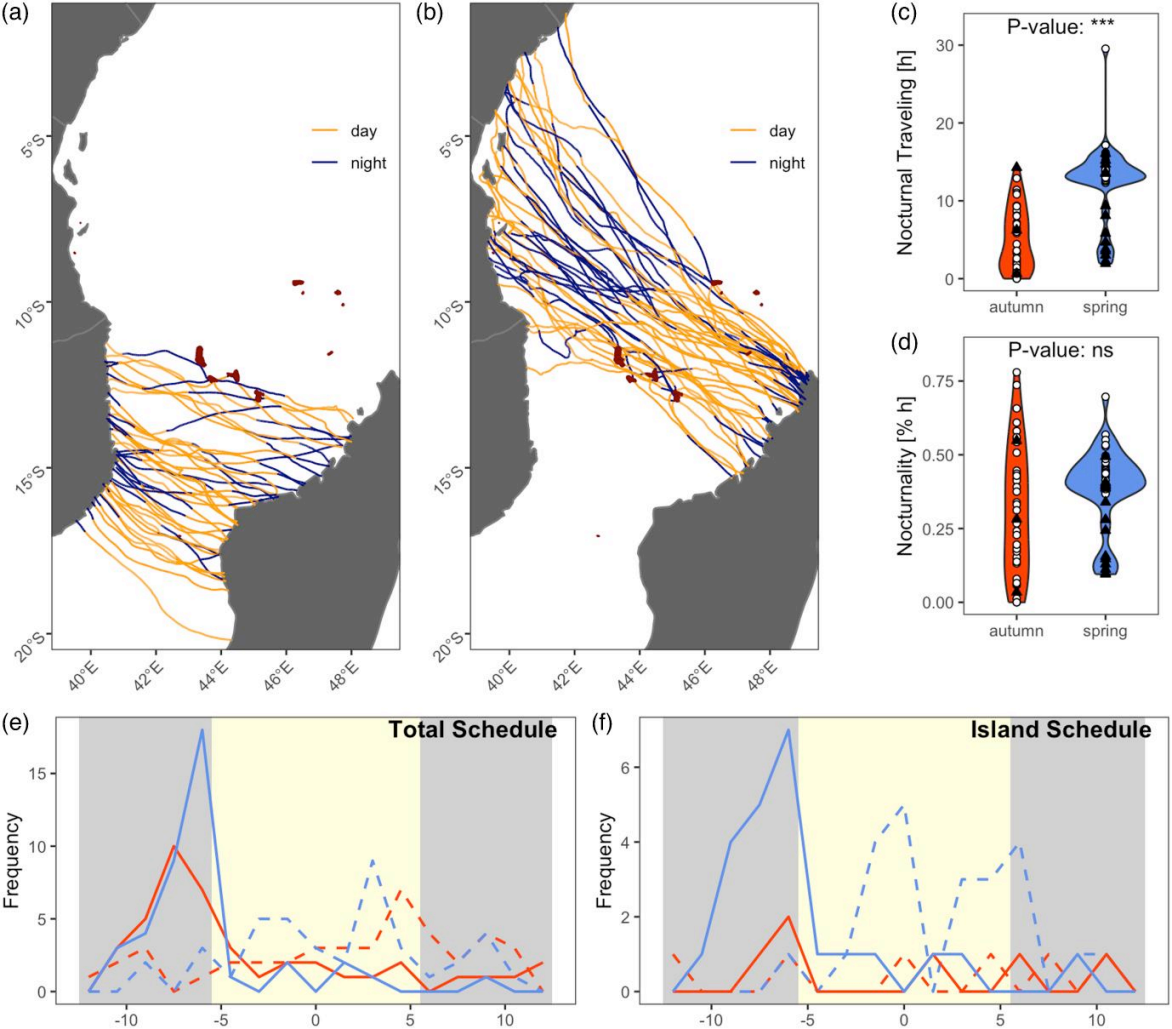
Nocturnality during ocean-crossings along with diel timing of take-offs and arrivals. (a) Autumn and (b) spring ocean-crossing routes indicating nocturnal (including twilight) and diurnal flight, as identified in the figure legend. (c) The number of nocturnal flight hours and (d) proportion of nocturnal flight hours, with lay-out analogous to panels Fig. 2C-I. (e–f) Frequency distributions of take-off times (solid lines) and arrival (dashed lines) times (e) at either end of the ocean-crossing and (f) on islands. X-axis shows solar time (ie hours vs solar noon). The background shading indicates the average daytime and night-time periods on the falcon's ocean-crossing dates.

Ocean-crossings involved substantial sections of nocturnal flight in both autumn (Fig. 3a) and spring (Fig. 3b). On average, falcons flew significantly more hours at night during the much longer spring crossings (Fig. 3c, Table S2). However, there was no significant difference in the average proportion of nocturnal flight time, and on average <50% of the total crossing was conducted at night in both seasons (Fig. 3d, Table S2). There was, however, much greater variation in the amount and proportion of nocturnal flight hours during autumn than in spring. In spring, nocturnal travel corresponded to approximately 30 to 50% of total crossing time (Fig. 3c, d). The notable exceptions were 2 cases where falcons reduced nocturnal travel time to ca. 3 to 4 h, ie < 20% of total crossing time, by overnighting on islands (Fig. 3c, d, note both “outlier” data points are black triangles).

In spring, initiation of ocean-crossings occurred within a narrow early-dawn window, both from Madagascar (Fig. 3e, solid blue line) and when departing from islands (Fig. 3f). In autumn, departure times were more broadly distributed across daylight hours (Fig. 3e, f). In both seasons, falcons completed sea-crossings (Fig. 3e, dashed lines) or reached islands (Fig. 3f, dashed lines) at spread out times throughout the daylight hours, and to a lesser extent at nighttime.

Trajectory speeds recorded on islands indicate that falcons were active during the day and resting at night (Figure S1a–d). When using an island in spring (typically in the 2nd half of the day), island-use events were characterized by falcons resting overnight (Figure S2e, f), resulting in a high proportion of time spent during nighttime hours. Earlier arrival times did not result in longer rest periods; indicating that falcons that reached islands during the day spent the rest of the daytime period patrolling or foraging rather than resting (Table S4).

### Seasonal differences in wind support and flight effort

The NOAA-NCEP Reanalysis II model indicates that, on average, winds opposed the falcons' travel direction in autumn (mean headwind: −0.5 ± 2.6 m/s), whereas moderate tailwind assistance prevailed in spring (mean tailwind: 3.8 ± 2.5 m/s). While we did not formally test whether falcons selected days with relatively favorable winds to initiate seasonal crossings, winds generally opposed the falcons travel direction on the great majority of days in autumn, and ocean-crossing dates were not clearly linked to rare windows of supportive winds (Figure S2a). By contrast, prevailing winds provided moderate-strong tailwind assistance along the falcons' mean flight direction on almost every day of the spring migration period (Figure S2b).

Despite the strong seasonal contrast in tail-/headwinds experienced over the ocean, falcons crossed the ocean at comparable ground speeds of 11.7 ± 2.0 m/s in autumn and 12.1 ± 1.5 m/s in spring. As a result, airspeeds were higher in autumn (13.2 ± 2.6 m/s) than in spring (8.8 ± 1.7 m/s). Integrated over the entire crossing, adverse autumn winds resulted in a net backward displacement of 93.0 ± 152.5 km, whereas spring winds resulted in a net forward displacement of 367.1 ± 193.4 km (Fig. 2c–e, Table S1). Consequently, the seasonal difference in “air distances” was much smaller than the seasonal difference in “ground distances” flown over the ocean. Nevertheless, falcons still achieved a 40% greater air distance (ie cumulative flight effort) over the ocean in spring (965.2 ± 192.8 km) than in autumn (717.4 ± 263.3 km).

### Wind effects on island use

**Figure 4:**
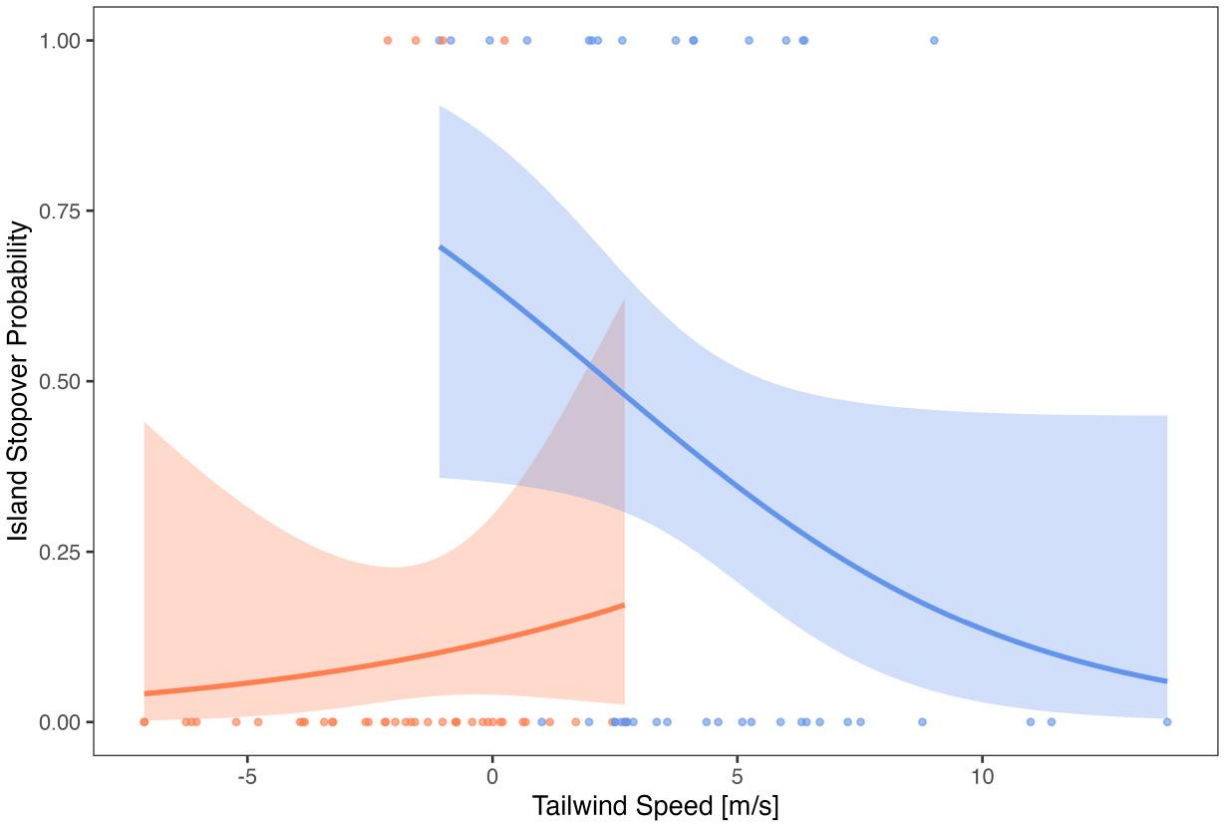
Logistic regressions for the effect of average tailwind strength during the first half of ocean-crossings on island stop-over probability.

We found evidence that island use was correlated with wind conditions experienced during the first half of the ocean-crossing in spring, but not in autumn (Fig. 4, Table S3). A single-effect logistic regression model did not reveal a significant effect of wind conditions on the probability of island use in either season (Fig. 4, Table S3). However, models that accounted for departure latitude showed that the probability of island use and time spent on islands significantly increased with weaker wind support in spring (Table S3). The effect of departure latitude is due to the fact that in autumn falcons did not use the San Juan island in the Mozambique channel, and only crossings that were initiated relatively far north on the East African coast were likely to include the Mayotte and Comoros Islands. In spring, falcons departing from the southern end of the crossing corridor were much more likely to encounter and use the Mayotte and Comoros islands than those departing from the northern end, which only rarely used the Aldabra group of the Seychelles (Table 2).

## Discussion

Using high-resolution movement data we were able to quantify how seasonal ocean-crossings of Eleonora's falcons compare in terms of flight effort. It was previously established that falcons' seasonal route choice allows them to reduce headwinds through adverse autumn wind fields and to maximize wind support through supportive spring wind fields (Mellone et al. 2011; Nourani et al. 2021; Vansteelant et al. 2021). Here we reveal that even while flying through the area with the strongest supportive winds over the Indian Ocean in spring, and through the area with weakest headwinds in autumn, falcons still cover a 40% greater air distance over the ocean in spring than in the majority of autumn crossings. The overall greater crossing effort in spring is associated with apparent energy-conserving behaviors, such as wind-dependent airspeed adjustments. Such airspeed adjustments allow falcons to reduce instantaneous energy expenditure, and to increase their potential flight time and range (Liechti 2006; Vansteelant et al. 2017b). Moreover, falcons stopped to rest on islands more often in spring. While this is partly due to more islands being available in the spring corridor. The probability of falcons using these islands was strongly influenced by departure positions from the mainland or Madagascar in autumn and spring, respectively, with an additional negative effect of tailwind support in spring. We interpret spring island visits as opportunistic stop-overs, not unlike wind-dependent use of “emergency stop-over sites” reported in other species (Shamoun-Baranes et al. 2010, 2017).

Seasonal and regional variations in the ground speeds of migratory birds are often explained by comparable variations in wind support (Liechti 2006; Vansteelant et al. 2015; Shamoun-Baranes et al. 2017). When considering the entire trans-African migration, this is also true for the Eleonora's falcons we studied here, which were previously shown to complete their ca. 20% longer spring migrations in the same amount of flight hours as in autumn, thanks largely to falcons attaining higher ground speeds in supportive winds in spring (Vansteelant et al. 2021). In this sense, it seems surprising that falcons achieved similar ground speeds in both seasons, despite large seasonal differences in wind support over the ocean. However, it has also been established that the relationship between tailwind support and ground speeds varies across different regions of the falcons' trans-African flyways (Vansteelant et al. 2021; Mellone et al. 2015; López-López et al. 2009). The ground speeds they achieve over the ocean are the highest they achieve anywhere in the flyway, including the autumn desert crossing where falcons enjoy even greater tailwind support than over the Indian Ocean in spring (Vansteelant et al. 2021). This can probably be explained by the consistent use of flapping flight over the ocean (Nourani et al. 2021), as opposed to frequent soaring-gliding flight over land. Moreover, our results enforce the notion that regional and seasonal variation in falcons' ground speeds are at least partly due to falcons adjusting airspeeds between energy- and time-minimizing optima depending on landscape and atmospheric contexts (Hedenström and Alerstam 1995; Harel et al. 2016; Vansteelant et al. 2017b). In fact, it appears that falcons flew near minimum-power airspeeds during spring crossing (ie minimizing fuel consumption per unit time) while flying closer to maximum-range airspeeds in autumn (ie minimizing fuel consumption per unit distance), and in both cases below maximum sustained flight speeds (Hedenström et al. 1999).

Migratory birds can make adaptive use of wind conditions in other ways that we have not tested here, for example by tuning the timing and altitude of flight to temporal and altitudinal variation in wind support (Shamoun-Baranes et al. 2017; Manola et al. 2020; Norevik et al. 2023). We know from previous work that the falcons we studied here occasionally stop-over for 1 to 5 days on the East African coast before initiating the ocean-crossing in autumn (Vansteelant et al. 2021). Furthermore, there are 3 cases of aborted ocean-crossings in our data (pers. obs.) and one in the study of Gschweng et al. (2008). This indicates that falcons sometimes do postpone or abort crossings in particularly adverse winds (Meyer et al. 2000; Santos et al. 2020). Nevertheless, our exploration of daily wind conditions in the falcons' seasonal departure areas suggests limited opportunities for falcons to select windows of supportive wind to cross the Mozambique channel in autumn, and little need for falcons to avoid windows of adverse winds in spring (Figure S1).

While previous tracking studies yielded anecdotal evidence of island use by ocean-crossing falcons (Gschweng et al. 2008; Hadjikyriakou et al. 2020), our analyses reveal that the use of remote islands is a common, although irregular and wind-dependent behavior among experienced Eleonora's falcons. The most used islands were Comore Grande (26% of falcons) and Moheli (21% of falcons) in the Comoros and the island of Mayotte (21% of falcons). The probability of falcons using these islands was partly correlated with latitude of departure, with falcons departing at the northern end of the autumn corridor, or the southern end of the spring corridor, being more likely to encounter these islands. However, not all falcons that passed close to these islands effectively used them, and we found a negative effect of wind support on island use in spring. Falcons also tended to stay on these islands longer in spring by extending their stay through the night to depart the next morning. Although we did not evaluate if staying on islands allows falcons to leave with better wind support the next day, we believe the main advantage of using islands is to sleep and recover in the middle of a protracted ocean-crossing.

The tendency of Eleonora's falcons to depart from islands at dawn in spring is consistent with their tendency to initiate sea-crossings in the early hours of dawn from Madagascar in spring. Falcons generally show a strong preference for diurnal migration, extending flights through the night mostly over inhospitable barriers (Strandberg et al. 2009; López-López et al. 2010; Lopez-Ricaurte et al. 2021; Vansteelant et al. 2021). The apparent tendency of Eleonora's falcons to complete as much of the Indian Ocean crossing as possible by day in spring could be due to the fact that winds tend to be weaker, while turbulence tends to be stronger over the ocean during the night than during the day (Nourani et al. 2021). Conversely, it is striking how falcons complete much of the autumn-crossings at night, considering they could complete most of their ca.12 h autumn crossings by day if they were to initiate crossings more consistently at dawn. Weaker winds at night (Nourani et al. 2021) could be a reason why some falcons conduct more than half of the Mozambique Channel crossing at night in adverse autumn wind fields.

Overall, our results suggest that remote islands provide stop-over opportunities to falcons crossing the ocean in spring, with a little more than half of birds using an island at least once during 1 to 4 yr of consecutive tracking. It is important to consider we only obtained ocean-crossing data for successful adult migrants, and it is still unclear whether mortality might be associated with inclement weather events over the Indian Ocean. Therefore, it also remains unclear to what extent these stop-over opportunities improve the survival and lifetime fitness of our study species. The southern and northern limit of the falcons' spring corridor, and the northern limit of their autumn corridor, was aligned with archipelagos in the Indian Ocean. Could it be that the falcons' spring ocean-crossing corridor is shaped in part by the availability of remote islands, in addition to being situated in the area with strongest supportive winds (Fig. 2)?

While it was previously established that our study species displays high flexibility (and low individual repeatability) in migratory route choice and timing over the Indian Ocean (Mellone et al. 2011; Vansteelant et al. 2023), we now revealed island use and flight speed adjustments as 2 additional highly flexible aspects of ocean-crossing behavior. There is a growing body of evidence suggesting that such behaviors are acquired through experiential and social learning in long-lived birds (Campioni et al. 2020; Sergio et al. 2022; Aikens et al. 2024; Brønnvik et al. 2024). Tracking of juvenile Eleonora's falcons has so far revealed that they respond differently to wind conditions than adults (Mellone et al. 2015), and that they often cross the Mozambique channel later and less efficiently than adults in autumn (Gschweng et al. 2008). In fact, some juveniles do not reach the Malagasy nonbreeding grounds at all on their first migration, spending their first nonbreeding season on mainland Africa instead (Gschweng et al. 2008; Mellone et al. 2013). It remains to be seen whether such individuals can learn their way to Madagascar, and the ocean-crossing skills this requires, later in life.

## Supplementary Material

arag094_Supplementary_Data

## Data Availability

Analyses reported in this article can be reproduced using the data provided by Chen et al. (2026). Raw tracking data are stored in the UvA-BiTS database (www.uva-bits.nl). Preprocessed data used in this study are available from DataverseNL (https://doi.org/10.34894/BZJAST). The code for reproducing our analyses is available in the GitHub repository (https://github.com/meixucm/eleonoras-falcon-ocean-crossing). This repository automatically installs all required packages and includes detailed instructions for reproducing the analyses and obtaining open-access third-party data.
