# Supplementary material for: Seasonal variation in trans-oceanic flight and island use in a migratory falcon
Source: Behav Ecol. 2026 Aug 3;37(5):arag094. doi: 10.1093/beheco/arag094 (PMC13487336; doi:10.1093/beheco/arag094)
Supplement: Supplementary file 1 [file arag094_supplementary_data.zip › Chen_et_al_BE_SupplementaryFigures_R1_Clean.pdf]

Supplementary Figures

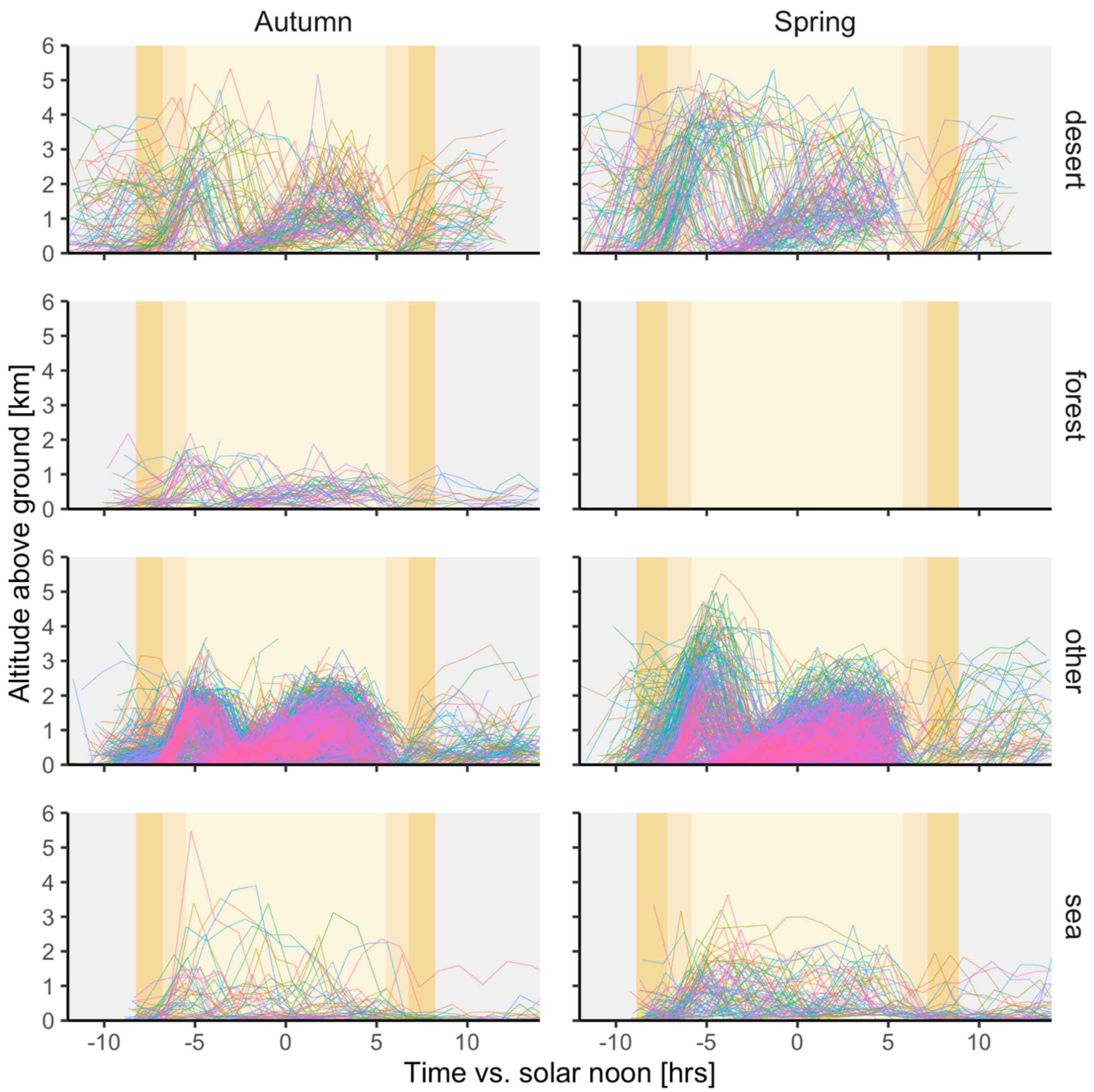

Figure S1. Altitude plots based on hourly data classified per region as in Vansteelant et al. (2021). Profiles show substantial differences in flight altitude between regions, and during sea-crossings flight altitudes are mostly between 100m to 1km above sea level, albeit with exceptions up to several km altitude, especially at the start of the sea-crossings when falcons could have climbed to substantial altitude over land before initiating a crossing. Due to computational limitations, we did not interpolate wind estimates to the specific altitude of the bird from different pressure levels. We instead used the 925mb pressure level as this best corresponds to the falcons' mean flight altitudes.

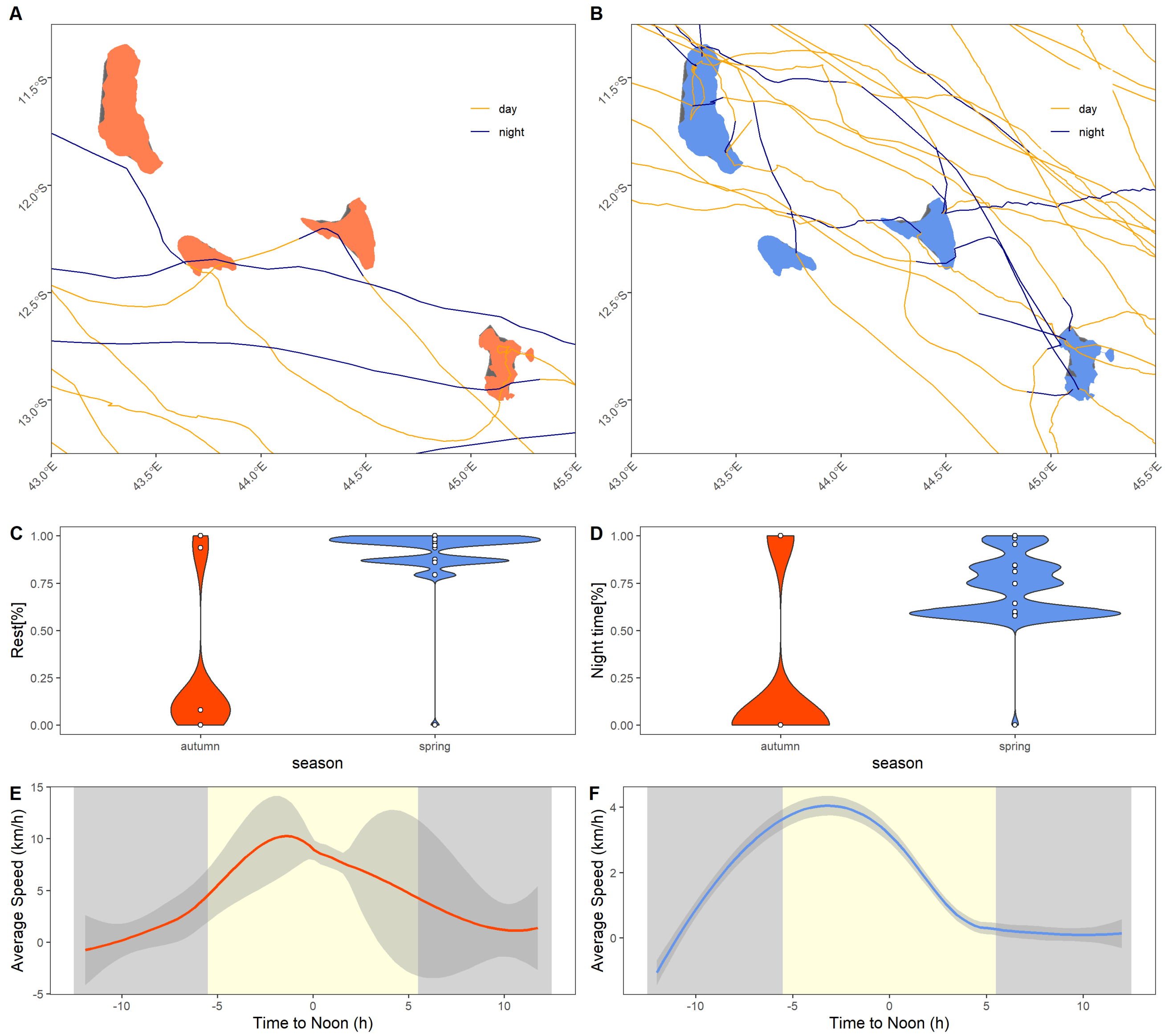

Figure S2. Exploring activity patterns of Eleonora's falcons on islands. (A,B) Detailed maps showing tracks passing over or near the Comoros and Mayotte in autumn (A) and spring (B), respectively. (C,D) Violin plots showing (C) the proportion of time a falcon spent resting during an island visit and (D) the proportion of island visits that occurred at night. (E,F) Average ground speeds of falcons recorded over islands in relation to time of day. Background shading indicates the average daytime period (light yellow) and nighttime period (light gray).

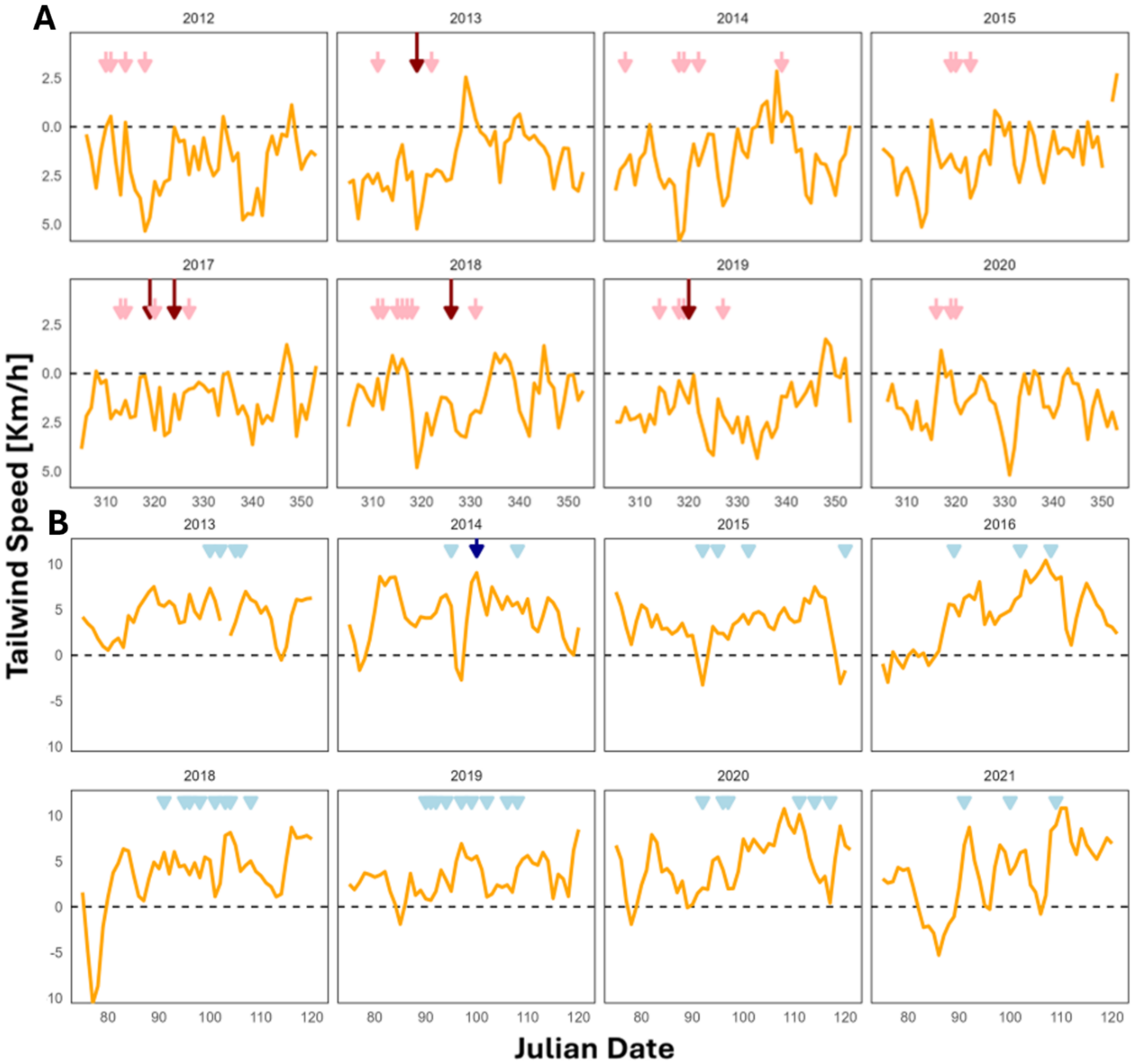

Figure S3. Dates of ocean-crossings by Eleonora's falcons in relation to daily tailwind conditions in the falcon's seasonal departure areas during (A) autumn and (B) spring, with each panel showing one of eight study years between 2012 to 2021. Orange lines indicate daily tailwinds across the falcons' seasonal departures areas, calculated relative to the falcons' average seasonal ocean-crossing direction. Coloured arrows indicate the dates on which crossings were recorded in autumn (red) and spring (blue). Lighter and shorter arrows indicate days on which one individual departed, whereas darker and longer arrows denote a higher number of departing individuals.
