# Supplementary material for: Seasonal variation in trans-oceanic flight and island use in a migratory falcon
Source: Behav Ecol. 2026 Aug 3;37(5):arag094. doi: 10.1093/beheco/arag094 (PMC13487336; doi:10.1093/beheco/arag094)
Supplement: Supplementary file 1 [file arag094_supplementary_data.zip › Chen_et_al_BE_SupplementaryTables_R1.pdf]

Table S1. Seasonal differences in ocean-crossing metrics (n = 83 crossings, 19 falcons), as estimated by generalized linear mixed models (GLMMs) with random intercepts per individual. Conditional R^2 could not be estimated for three response variables due to singular model fit, indicating that the inclusion of random intercepts resulted in over-fitting. In such cases we instead used standard linear models and only R^2 values are reported (models C, F, K). For the variable ‘island count’ (model F) we applied zero-inflated Poisson (ZIP).

Figure	Response	R^2cond	R^2mar/ R^2	Predictor	Estimate	Std. Error	df	t/z value	Pr(>|t/z|)
Fig.1C	Distance	NA	0.81	Autumn	624.4	26.88	81	23.23	**1.47E-37**
				Spring	707.94	38.25	81	18.51	**7.88E-31**
Fig.1D	Duration	0.7	0.69	Autumn	15.34	0.84	49.95	18.24	**9.77E-24**
				Spring	15.68	1.15	69.14	13.68	**2.42E-21**
Fig.1E	Duration Island	0.21	0.13	Autumn	0.38	1.13	45.54	0.34	7.35E-01
				Spring	5.38	1.44	68.31	3.73	**3.93E-04**
Fig.1F	Islands Count	NA	0.41	Autumn	-1.45	0.52	79	-2.76	**5.86E-03**
				Spring	1.32	0.49	79	2.67	**7.66E-03**
Fig.1G	Tail Wind speed	0.43	0.42	Autumn	-0.47	0.4	48.72	-1.18	2.43E-01
				Spring	4.29	0.55	68.21	7.77	**5.53E-11**
Fig.1H	Ground Speed	0.15	0.01	Autumn	42.29	1.09	42.7	38.98	**5.15E-35**
				Spring	1.05	1.3	68.35	0.81	4.22E-01
Fig.1I	Air Speed	0.52	0.51	Autumn	47.38	1.24	50.26	38.26	**7.80E-39**
				Spring	-15.89	1.71	68.92	-9.28	**9.54E-14**
Fig.1J	Wind Displacement	0.7	0.64	Autumn	-91.49	30.13	37.67	-3.04	**4.32E-03**
				Spring	459.64	34.51	66.41	13.32	**1.99E-20**
Fig.1K	Air Distance	NA	0.23	Autumn	717.41	35.67	81	20.11	**3.09E-33**
				Spring	247.83	50.76	81	4.88	**5.17E-06**

Table S2. Seasonal nocturnality differences in ocean-crossing metrics (n = 83 crossings, 19 falcons), as estimated by generalized linear mixed models (GLMMs) with random intercepts per individual.

Figure	Response	R^2cond	R^2mar	Predictor	Estimate	Std. Error	df	t/z value	Pr(>|t/z|)
Fig.3D	Nocturnality [%h]	0.06	0.04	Autumn	0.31	0.03	50.01	11.1	**4.31E-15**
				Spring	0.08	0.04	68.75	1.94	5.69E-02
Fig.3C	Nocturnal travel time [h]	0.39	0.39	Intercept	5.12	0.7	55.61	7.26	**1.32E-09**
				Spring	7.19	1	71.18	7.23	**4.47E-10**

Table S3. Results of generalized linear models (GLMs) testing the effects of wind speed during ocean crossings and the start latitude of ocean-crossings on the number of islands used by Eleonora’s falcons during autumn and spring. Models were fitted using a Poisson error distribution with a log-link. We used quasi-AIC (QAIC) to identify the best model for each season (bolded QAIC). Pseudo-R^2 (McFadden's) are provided to assess explanatory power of the models.

response	Season	Estimate	Std. Error	z value	Pr (>|z|)	Predictor	R^2	QAIC
Island use	Spring	-0.18	0.34	-0.52	6.03E-01	Intercept	0.07	75.49
		-0.10	0.07	-1.35	1.77E-01	Tailwind		
		-7.95	2.77	-2.87	**4.09E-03**	Intercept	0.24	76.31
		-0.54	0.20	-2.73	**6.37E-03**	Start latitude		
		0.89	4.80	0.19	8.52E-01	Intercept	0.39	**58.22**
		0.09	0.36	0.27	7.90E-01	Start latitude		
		-2.48	1.20	-2.07	**3.84E-02**	Tailwind		
		-0.18	0.09	-2.02	**4.34E-02**	Start latitude*Tailwind		
		-7.14	2.82	-2.53	**1.14E-02**	Intercept	0.27	76.08
		-0.51	0.20	-2.55	**1.09E-02**	Start latitude		
		-0.08	0.08	-1.00	3.16E-01	Tailwind		
	Autumn	-1.68	0.44	-3.82	**1.34E-04**	Intercept	0.06	**30.44**
		0.19	0.19	1.04	2.99E-01	Tailwind		
		21.23	7.90	2.69	7.18E-03	Intercept	0.74	71.84
		1.83	0.67	2.74	6.22E-03	Start latitude		
		20.02	7.18	2.79	5.29E-03	Intercept	0.78	73.93
		1.70	0.61	2.77	5.54E-03	Start latitude		
		1.49	3.35	0.44	6.57E-01	Tailwind		
		0.10	0.27	0.38	7.03E-01	Start latitude*Tailwind		
		19.38	6.71	2.89	3.87E-03	Intercept	0.77	72.30
		1.65	0.57	2.89	**3.88E-03**	Start latitude		
		0.22	0.22	0.99	3.24E-01	Tailwind		

Table S4. Results of the GLM examining whether the proportion of resting time on islands was correlated with the amount of time spent on an island at night, by day, or the total duration of island visits. We assumed a beta error distribution using a logit link function. Each model includes only one predictor. For clarity, intercepts are reported only for the null models. R^2 values reflect the variance explained by the fixed effects.

Response	Season	Predictor	Estimate	Std. Error	z value	Pr(>|z|)	R^2	AIC
rest [%]	spring	Intercept (null model)	1.88	0.03	64.87	0.00E+00	0.00	-6872.04
		Intercept	-0.82	0.07	-11.29	**1.55E-29**	0.54	**-7476.03**
		nighttime duration	0.29	0.01	35.81	**7.22E-281**		
		Intercept	2.14	0.04	50.68	0.00E+00	0.12	-6943.05
		daytime duration	-0.06	0.01	-8.59	**8.74E-18**		
		Intercept	2.33	0.12	19.23	2.09E-82	0.05	-6884.88
		total duration	-0.03	0.01	-3.91	**9.36E-05**		
	autumn	Intercept (null model)	-0.44	0.09	-4.98	6.35E-07	0.00	-176.00
		Intercept	-2.10	0.08	-27.76	1.28E-169	0.98	-406.58
		nighttime duration	1.01	0.06	17.67	**6.83E-70**		
		Intercept	2.48	0.14	17.96	4.20E-72	0.93	**-543.43**
		daytime duration	-1.87	0.07	-26.20	**2.66E-151**		
		Intercept	-1.95	0.15	-13.03	8.35E-39	0.83	-300.54
		total duration	0.38	0.04	10.49	**9.75E-26**		
